# Assessing the geographic scale of genetic population management with microsatellites and introns in the clam *Ruditapes decussatus*

**DOI:** 10.1002/ece3.2052

**Published:** 2016-04-15

**Authors:** Alberto Arias‐Pérez, David Cordero, Yaisel Borrell, Jose Antonio Sánchez, Gloria Blanco, Ruth Freire, Ana Insua, Carlos Saavedra

**Affiliations:** ^1^ Departamento de Bioloxía Celular e MolecularUniversidade da CoruñaA Zapateira s/n15071A CoruñaSpain; ^2^ Instituto de Acuicultura Torre de la SalConsejo Superior de Investigaciones Científicas12595Ribera de Cabanes (Castellón)Spain; ^3^ Departamento de Biología FuncionalUniversidad de Oviedo33006OviedoSpain

**Keywords:** Bivalves, genetic differentiation, genetic variability, marine genetic resources, population genetics, *Ruditapes decussatus*

## Abstract

The clam *Ruditapes decussatus* is commercially important in southwestern Europe, suffering from population decline and hybridization with exotic Manila clam (*R. philippinarum*). Previous studies with intronic markers showed a genetic subdivision of the species in three races (Atlantic, West Mediterranean, and Adriatic‐Aegean). However, detailed population genetic studies to help management of the main production areas in the southwest of Europe are missing. We have analyzed eight Atlantic and two Mediterranean populations from the Spanish coasts using 14 microsatellites and six intronic markers. Microsatellites confirmed the Atlantic and West Mediterranean races detected with introns and showed that genetic variability was higher in Mediterranean than in Atlantic populations. Both marker types showed that genetic differentiation of Atlantic populations was low and indicated that populations could be managed at the regional level in the case of Cantabrian and Gulf of Cadiz areas, but not in the case of Rias Baixas and the Mediterranean. This study shows the interest of including different types of markers in studies of genetic population structure of marine organisms.

## Introduction

The grooved carpet‐shell clam *Ruditapes decussatus* is an infaunal bivalve that lives in sandy‐muddy bottoms of estuaries, lagoons, and coastal flats along the Mediterranean Sea and the northeast Atlantic, from Senegal to Norway (Fischer‐Piette and Métivier 1971). The species is considered a food delicacy in southern Europe, and it is fished in France, Spain, Portugal, Italy, and in other Mediterranean countries. The global production was 5912 T in 2013 according to FAO (www.fao.org). Commercial exploitation is carried out through traditional methods based on collection by hand during low tides, or from small boats using rakes. The high prices that clams can reach in the market have led to intensive exploitation in several areas, and natural European populations have declined in many of the places where the species used to be common in the past. *R. decussatus* is facing also the spread of the Manila clam (*Ruditapes philippinarum*), which was introduced in Europe in the early 1970s to respond to the increasing demand of clams and to some limitations of carpet‐shell clams to cover this demand (Flassch and Leborgne 1992; Paesanti and Pellizzato 2000; Breber 2002). The carpet‐shell has been replaced by Manila clam in some areas, but it is not clear whether this replacement has been due to competence or to other factors (Flassch and Leborgne 1992; Jensen et al. 2004; Pranovi et al. 2006; Juanes et al. 2012; Bidegain and Juanes 2013). However, clear evidence of hybridization and introgression of Manila clam genes in *R. decussatus* has been reported in some localities, although in low rates (Hurtado et al. 2011; Habtemariam et al. 2015).

At present, there is no general management system that covers all the grooved carpet‐shell clam populations. Management practices rather vary across countries and regions, with lack of management across long coastal areas and intensive management in others. A common practice to recover exhausted natural beds of grooved carpet‐shell clams in many managed localities has been the release of spat collected in distant areas or, more frequently, obtained in breeding facilities (hatcheries) (Walne 1970; Helm and Pellizzato 1990; Jones et al. 1993; Passamonti et al. 1997; Turolla 2008).

To help designing restocking programs and managing strategies, there has been an interest in obtaining basic population genetic data. Several studies on the population genetics of *R. decussatus* have appeared along the last 25 years (Jarne et al. 1988; Borsa et al. 1991, 1994; Jordaens et al. 2000; Cordero et al. 2008, 2014; Gharbi et al. 2010, 2011; Pereira et al. 2011; Borrell et al. 2014; Habtemariam et al. 2015). However, only two studies have sampled a significant number of populations (Borsa et al. 1994; Cordero et al. 2014). Surprisingly, the two studies gave somewhat different results. Borsa et al. (1994) studied six enzyme polymorphisms by starch gel electrophoresis in five populations across the Mediterranean and one in southern Portugal and found overall levels of genetic variability which were comparable to other bivalve species and very low population differentiation (*F*_ST_ = 0.015). Borsa et al. (1994) also studied the genetic variability at a smaller scale in the coastal lagoons of southeast France and found no statistically significant genetic differences between lagoons, between sites within lagoons, or between temporal samples within sites.

In another study, Cordero et al. (2014) analyzed 11 populations ranging from Atlantic France to Turkey with six markers based on restriction fragment length polymorphism of introns (iRFLP) and partial sequences of the mitochondrial gene COI and of two introns. Unlike Borsa et al. (1994), they found a clear subdivision in three groups of populations or races: Atlantic populations (ATL), Mediterranean populations plus Tunisia (WMED), and Adriatic and Aegean populations (AEGAD). Moderate average genetic differentiation among populations was found (*F*_ST_ = 0.134), with high values at some loci (*F*_ST_ > 0.2). Differences between populations within each of the three races were also significant. Finally, the mtDNA marker showed a phylogenetic break located at the transition from the W Mediterranean Sea to the Adriatic and Aegean seas, which was corroborated by sequencing the most variable iRFLP markers.

In the present paper, we report the results of a study of 10 carpet‐shell clam populations from the coasts of Spain with the same iRFLP markers used by Cordero et al. (2014) and with 14 microsatellites recently developed for this species (Borrell et al. 2014). Our main goal is to study the genetic structure of the Atlantic populations of the carpet‐shell clam. The European Atlantic coast contains the most intensively exploited populations of carpet‐shell clam in Europe. In spite of this, only as many as four samples from these coasts were included in the studies of Borsa et al. (1994) and Cordero et al. (2014). Therefore, a specific study of a larger number of populations in the region would provide data which would be especially valuable for the management of the species. Specifically, previous studies suggested that large coastal areas could be genetically homogeneous but also suggested some regional subdivision, two aspects that need more detailed examination. Microsatellite markers are the tools of choice for this kind of study, but introns also showed high potential to detect population subdivision in the study of Cordero et al. (2014). On the other hand, microsatellites usually provide more accurate estimates of several population genetic parameters such as heterozygosity and inbreeding rates due to their higher number of alleles. Therefore, the combination of the two marker types is expected to provide a rich data set for clam genetic population management.

In addition, we aim to clarify the picture of the distribution of genetic variability inferred from previous studies in the range of the carpet‐shell clam. The differences between the allozyme study of Borsa et al. (1994) and the iRFLP study of Cordero et al. (2014) can be due to different causes such as small number of genes sampled, differences in mutation rates, or natural selection acting on one or both marker types (Avise 2004). Since neutrality is a basic assumption of population genetic studies that use molecular markers, excluding or confirming this possibility is of special interest in the case of introns because they have provided the largest data set obtained so far in *R. decussatus*. For this purpose, we scored microsatell‐ites and introns in the same samples. Microsatellites are usually considered strictly neutral markers, because the overwhelming majority is located in noncoding intergenic genome regions (Chistiakov et al. 2006). The observation of a high similarity between microsatellites and the iRFLPs in the patterns of geographic variation would support a neutral explanation for the patterns observed in the latter.

## Materials and Methods

### Sample collection, DNA extraction, and genotyping

**Figure 1:**
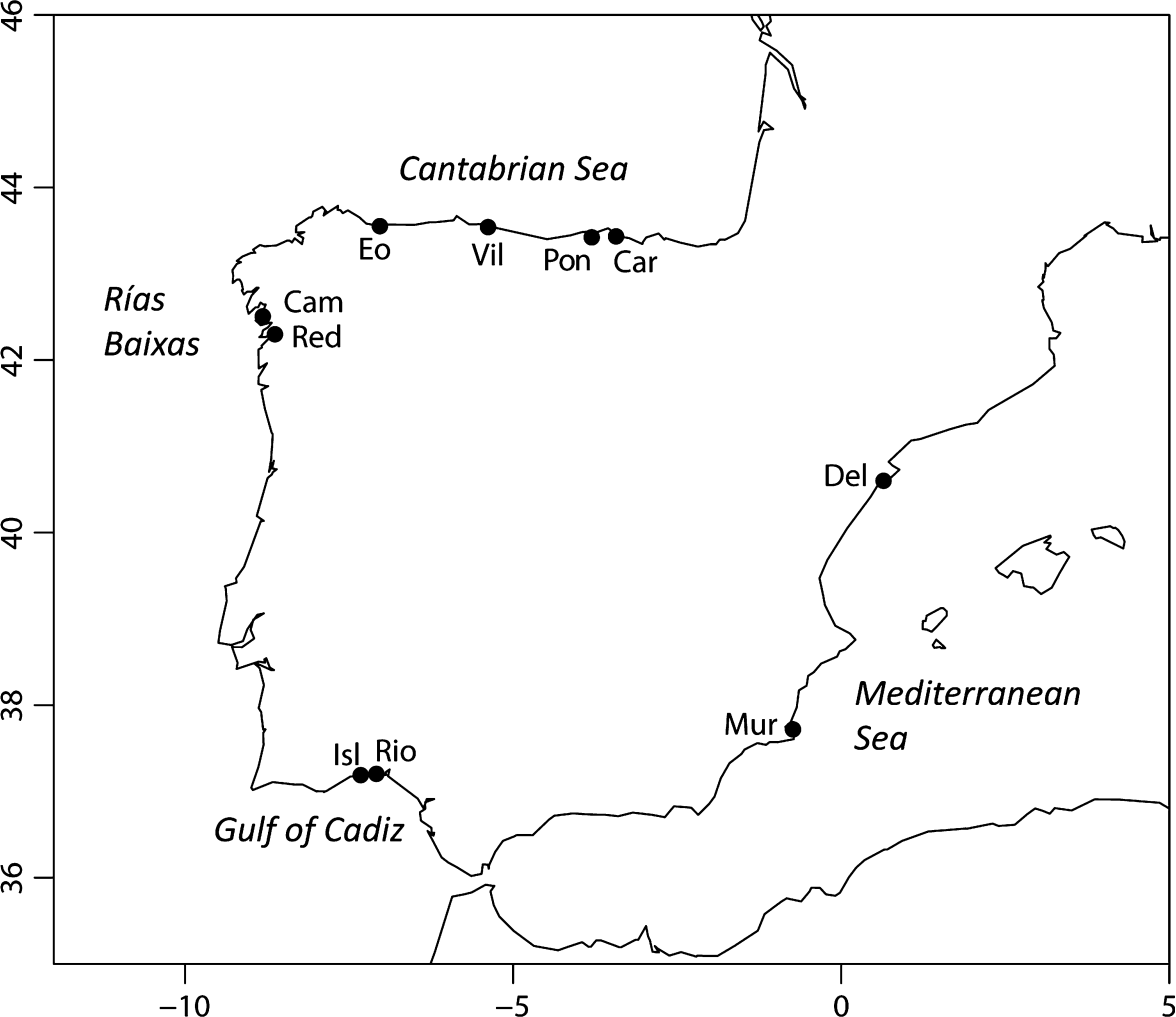
Map showing the localities sampled in this study. Car: Carasa; Pon: Pontejos; Vil: Villaviciosa; Eo: Ría del Eo; Cam: Cambados; Red: Redondela; Isl: Isla Cristina; Rio: Río Piedras; Mur: Murcia; Del: Ebro Delta.

A total of 513 individuals were collected from 10 Spanish natural populations distributed in four coastal regions: Cantabrian Sea, Rías Baixas, Gulf of Cadiz, and Mediterranean Sea (Fig. 1). Data for two samples, Eo and Vil, were the same used in Borrell et al. (2014). Genomic DNA was obtained from a small piece of adductor muscle using the Zymobead TM Genomic DNA Kit (Zymo Research Corp., Irvine, CA), the method of Fernández‐Tajes and Méndez (2007), or by boiling during 20 min in a 10% preparation of the cation exchange resin Chelex 100, 200–400 mesh (Bio‐Rad, Hercules, CA).

Six nuclear intronic regions described in previous studies (Cordero et al. 2008, 2014) were amplified by PCR (*Ech*,* Fas*,* Tbp*,* Trdmt*,* Srp54*, and *Ubc*). Genotyping of *Tbp* was based on its length polymorphism and was carried out by running the PCR products in a 2% agarose gel and photographed under UV light exposure. Genotyping of the five remaining markers was carried out by scoring their restriction fragment length polymorphism (RFLP) in 1.5% agarose gels under UV light. Specific conditions are explained in detail in Cordero et al. 2008.

Fourteen microsatellite loci arranged into two multiplex PCRs (RdMTP‐1 and RdMTP‐2) were genotyped as described in Borrell et al. (2014). Individuals whose genotype remained uncertain after scoring by two observers were discarded or regenotyped. Replicated samples were used to compute the error rate, expressed as the number of incorrect genotypes divided by the number of repeated reactions. The overall genotyping error rate per reaction was 0.019 (10 mistyped reactions of 678), being in the range reported by other studies (see Hoffman and Amos 2005, and references therein).

### Data analysis

Allelic and genotypic frequencies were calculated for each population and locus. Those individuals that did not amplify in more than two intronic loci were eliminated from the analysis. The unbiased estimate of expected heterozygosity (Nei 1978) and the mean allele number by locus were calculated for introns to measure the extent of genetic diversity, both with the software Arlequin v.3.0 (Excoffier et al. 2005). For microsatellites, basic data analysis was carried out following Arias et al. (2010) and Arias‐Pérez et al. (2012). The number of alleles and the observed and expected heterozygosities were obtained with Genetix v. 4.05.2 (Belkhir et al. 2004). Allelic richness, a measure of the number of alleles independent of sample size, per locus, locality, and overall was computed with *F*_stat_ v. 2.9.3 (Goudet 2001).

A Friedman test was conducted on expected heterozygosity (introns and microsatellites), number of alleles (introns), and allelic richness (microsatellites) with R software (R Core Team 2015) to check for differences among localities. When the tests were significant, a *post hoc* analysis based on Wilcoxon–Nemenyi–McDonald–Thompson procedure (Hollander and Wolfe 1999) was carried out using an R function (Galili 2010).

Deviations from Hardy–Weinberg equilibrium (HWE) at each locus and population were measured with the *F*_*IS*_ statistic (Weir and Cockerham 1984) and their significances were determined by means of exact tests (Raymond and Rousset 1995). Exact *P*‐values were calculated by the Markov chain method with 10,000 dememorization steps, 20 batches, and 5000 iterations per batch for introns and 10,000 dememorizations, 5000 batches, and 10,000 iterations per batch for microsatellites. A global *F*_IS_ across all loci and populations was obtained by the Fisher method (Sokal and Rohlf 1999). Linkage disequilibrium between pairs of loci at each population was tested by a *G*‐test computed using a Markov chain algorithm (Raymond and Rousset 1995) with 10,000 dememorizations, 100 batches and 5000 iterations per batch for introns and 10,000 dememorizations, 5000 batches, and 10,000 iterations per batch for microsatellites. Sequential Bonferroni correction was applied to *P*‐values of both tests (Rice 1989). All these calculations were carried out with Genepop v. 4.2 software (Rousset 2008).

Null allele frequencies at introns were calculated with the program INEst (Chybycki and Burczyc 2009) under the random mutation model (Kalinowski and Taper 2006; Chapuis and Estoup 2007) when necessary. Microsatellite data were examined for evidence of null alleles, allele dropout, or stuttering with the program Micro‐Checker (Van Oosterhout et al. 2004). Null allele frequency was estimated using the first method of Brookfield (1996). When multiple tests were performed, the significance values were adjusted using the sequential Bonferroni correction (Rice 1989).

To ensure that the markers employed in this study had adequate statistical power for detecting population structure, we used a simulation procedure implemented in POWSIM 4.1 (Ryman and Palm 2006). The analysis was carried out employing the overall allele frequencies of intron and microsatellite markers for all samples. Chi‐square and Fisher's exact tests were used to test the null hypothesis of identical allele frequencies. Different combinations of effective population size (10,000, 5000, 1000 and 500) and generations of drift were used to simulate various *F*_ST_ values. The power analysis showed alpha values reasonably close to the 5% value for both chi‐square and Fisher's exact tests. For microsatellite and intron markers, a *F*_ST_ value of 0.005 and 0.01 had a probability >=99% of being detected, respectively. This suggests that the markers employed here offer enough resolution to detect low levels of genetic structure. Genetic differentiation among populations was studied with three methods. First, the *F*_ST_ statistics, which measures the standardized variance of allelic frequencies in a group of samples (Weir and Cockerham 1984), was estimated in several ways. *F*_ST_ was computed for all populations, groups of populations, and population pairs. The significances of these estimates were obtained in Arlequin (Excoffier et al. 2005) for introns and in Genetix for microsatellites with 30,000 and 10,000 permutations, respectively. As the *F*_ST_ value is highly dependent on the level of genetic variation, making it difficult to interpret and compare the level of genetic differentiation between loci and studies, a standardized value (*F*_ST_ *’)* was calculated. Moreover, an overall analysis of genetic differentiation was carried out through hierarchical *F*_ST_ *’* statistics with the software GenoDive, v. 2.0b23 (Meirmans and van Tienderen 2004).

Second, genetic distances were computed for all pairs of populations, Nei distances (1972) for intron data, and Reynolds's genetic distance (Reynolds et al. 1983) for microsatellites. A tree was constructed using the neighbor‐joining method (Saitou and Nei 1987) using Phylip software 3.695 (Felsenstein 1993) and visualized using TreeView v. 1.6.6 (Page 1996). The robustness of the nodes was assessed by bootstrapping (10,000 times).

Third, a Bayesian clustering analysis was carried out with the software STRUCTURE, version 2.3.4 (Pritchard et al. 2000). The genotypic data of populations was analyzed under a clustering model of admixture of individuals with correlated allele frequencies among populations. The information of the sample locations was introduced as a prior. Analysis of intronic data includes modeling of clusters from *K* = 1 to *K* = 11 with 10 replicates of 200 000 iterations with previous burn‐in of 200 000 each one. Microsatellites were modeled for *K* values between 1 and 10, 20 runs each, and 100,000 burn‐in followed by 500,000 iteration steps. The best *K* was determined by looking for the maximum posterior probabilities differences (∆*K*) of contiguous *K* (Evanno et al. 2005), computed using Structure Harvester (Earl and von Holdt 2012). To search for optimal alignments of replicate clusters analysis and visualize individuals’ assignment, CLUMPP (Jakobsson and Rosenberg 2007) and Distruct (Rosenberg 2004) programs were used, respectively.

Additionally, we obtained the overall *R* ^2^ statistic for each clustering analysis (*K*) and each kind of marker with the software Obstruct (Gayevskiy et al. 2014). This statistic can be calculated from the average compilation of the Structure runs obtained with CLUMPP and permits us to objectively compare levels of structure among different data sets. Overall *R* ^2^ statistic gives us a measure of the correlation found between the inferred populations and the predefined populations. Statistical significance was obtained by 10,000 permutations of the assignments to ancestor profiles and the calculation of *R* ^2^ for each swap.

The contribution of each allele to the differentiation between clusters was calculated by computing the difference in allele frequencies between all pairs of clusters. These values were then represented graphically in a radar chart created with Excel.

To test for isolation by distance (Slatkin 1993; Rousset 1997), a Mantel test was performed in IBDWS v. 3.23 (Jensen et al. 2005) using 10,000 randomizations. The genetic differentiation between localities was measured as *F*_ST_/(1−*F*_ST_) and the geographic distance (km) as the coastline distance between sample locations.

The presence of loci affected by natural selection was tested by means of a Bayesian approximation that calculates departures from neutrality by studying the distributions of *F*_ST_ values with the program BayeScan v. 2.1 (Foll and Gaggiotti 2008). This method incorporates levels of uncertainty in allele frequencies, effective population size, and immigration rate among populations and also corrects for small sample sizes, and has been shown to incur in lower type I error rate than other selection tests (Garcia‐Figueroa et al. 2010; Narum and Hess 2011). Departures from neutrality are indicated by means of a locus specific component, the alpha component of the selective model. When significantly positive, alpha would suggest directional selection and would indicate balancing selection when negative values were obtained. We chose an odd prior of 100 for the neutral model, and a FDR of 5%.

## Results

### Genetic variation

#### Introns

Allele frequencies and other statistics are given in Appendix 1. The number of alleles per locus varied from 2 to 4. The mean allele number across populations was lowest at Red (2.3) and highest at Vil, Eo and Del (2.7). Heterozygosities by locality ranged from 0.353 to 0.482 (*H*_*e*_) and from 0.260 to 0.486 (*H*_*o*_). One private allele at low frequency was found at marker *Tbp* in Eo. No significant differences in allele number or expected heterozygosity among localities were detected (Friedman chi‐square, *P *>* *0.05).

Two intronic loci showed positive significant deviations from HWE after sequential Bonferroni correction (Appendix 1). Significant *F*_IS_ were always positive and varied between 0.060 and 1.000. *Ech* deviated in eight localities and *Srp54* in five. Null allele estimates for *Ech* resulted in frequencies higher than 0.2 at all localities except Del (0.064), while no null alleles were detected for locus *Srp54* at those localities with significant deviations. Therefore, we considered null alleles only at *Ech*.

#### Microsatellites

Genetic variation statistics by locus, locality, and overall are collected in Appendix 2. The number of alleles per locus ranged between 6 and 18 and the allelic richness between 3.9 and 12.9. Across localities, the allelic richness and the mean number of alleles varied between 6.9 and 8.2, and between 7.3 and 8.7, respectively. In both cases, the lowest value was observed in Cam and the highest one in Del. The heterozygosity by locality was 0.613–0.697 (*H*_*o*_) and 0.633–0.736 (*H*_*e*_). Private alleles (1–7) were present in all localities, except at Pon, Vil and Rio. They were always at low frequencies (<2.5%), except one locus at Mur (frequency: 0.163, locus RdATC‐238). The Friedman test detected significant differences in allelic richness (Friedman chi‐square = 20.066, df = 9, *P *=* *0.018) and expected heterozygosity (Friedman chi‐square = 21.522, df = 9, *P *=* *0.011) among localities. A *post hoc* analysis showed significant differences between Cam and Mur for allelic richness and between Mur and three localities (Cam, Red, and Rio) for expected heterozygosity.

Deviations from HWE were found in only four cases after sequential Bonferroni correction: the locus RdATC‐238 departing in two localities and the loci RdATC‐022 and RdATC‐219 in one. Deviations were always caused by a heterozygote deficit (*F*_IS_ ≥ 0.258). Results from Micro‐Checker analysis suggested the presence of null alleles in the four cases (frequency 0.112–0.227), but only one of them showed null allele frequencies over 0.200 (RdATC‐238 in Red). Of the tests performed to detect linkage disequilibrium only, the comparisons involving RdATC‐022 and RdATC‐199 were significant after sequential Bonferroni correction. This suggests that these loci might be closely linked and that they cannot be treated as independent variables.

### Population genetic differentiation

#### Introns

The overall multilocus estimate for *F*_ST_ was significan‐tly different from zero (*F*_ST_ = 0.088, *P* ≤ 0.001, *F*_ST_ *’ *= 0.161), indicating the existence of genetic differentiation among samples. Values of *F*_ST_ across markers (Table 1) ranged from 0.010 (*Ubc*) to 0.194 (*Trdmt*), and *F*_ST_ *’* ranged from 0.016 to 0.337. *F*_ST_ for Atlantic populations alone was 0.051.

**Table 1:** Overall *F*_ST_ per locus, standardized *F*_ST_ (*F*_ST_’), and results of the test for *F*_ST_ outliers of Foll and Gaggiotti (2008) (alpha and *q*‐values), for intronic and microsatellite markers

Locus	*F*_ST_	*P*‐value	*F*_ST_’	Alpha	*q*‐value
Intron RFLPs					
*Ech*	0.112	<0.0001	0.278	−0.2542	0.444
*Fas*	0.069	<0.0001	0.087	−0.0034	0.757
*SRP54*	0.024	<=0.0014	0.079	−1.3567	0.080
*TBP*	0.096	<0.0001	0.166	−0.0210	0.698
*Trdmt*	0.194	<0.0001	0.337	−0.0002	0.796
*Ubc*	0.010	<=0.0825	0.016	−0.1167	0.605
Microsatellites					
*RdATC‐1.34*	0.029	<0.0001	0.154	−1.4570	0.006
*RdATC‐1.79*	0.015	<0.0001	0.092	−1.8325	0.000
*RdATC‐125*	0.034	<0.0001	0.110	−0.0219	0.395
*RdATC‐177*	0.020	<0.0001	0.155	−1.7642	0.000
*RdATC‐185*	0.021	<0.0001	0.033	−0.5009	0.236
*RdATC‐199*	0.020	<0.0001	0.077	−0.4376	0.176
*RdATC‐212*	0.040	<0.0001	0.153	−0.8731	0.100
*RdATC‐215*	0.014	<0.0001	0.121	−1.1025	0.012
*RdATC‐219*	0.033	<0.0001	0.059	−0.0024	0.486
*RdATC‐223*	0.080	<=0.023	0.013	−0.2474	0.337
*RdATC‐238*	0.012	<0.0001	0.084	−1.0316	0.050
*RdATC‐263*	0.098	<0.0001	0.261	0.0139	0.444
*RdATC‐28b*	0.030	<0.0001	0.050	−0.4832	0.285

Pairwise multilocus *F*_ST_ (Table 2) ranged from −0.004 (Eo‐Vil) to 0.242 (Del‐Red), and it was significant after sequential Bonferroni correction in 22 cases. The four Cantabrian samples showed no significant pairwise *F*_ST_ comparisons, thereby forming a homogeneous group. The same happened with Isl and Rio. Rías Baixas populations (Cam and Red) showed a significant result (but not after the Bonferroni sequential procedure), and in this case, the *F*_ST_ value (0.025) was relatively high despite their proximity. The highest pairwise *F*_ST_ values were obtained when comparing the Mediterranean populations with the Atlantic.

**Table 2:** *F*_ST_ (above the diagonal) and *F*_ST_’ (below the diagonal) between pairs of localities of *Ruditapes decussatus*, estimated from intron RFLP markers

	Car	Pon	Vil	Eo	Cam	Red	Isl	Rio	Mur	Del
Car		0.017^a^	0.009	0.007	0.078^b^	0.115^b^	0.020^a^	0.02	NA	0.121^b^
Pon	0.032		0.009	0.011	0.100^b^	0.149^b^	0.038^b^	0.033^a^	NA	0.173^b^
Vil	0.016	0.016		−0.004	0.091^b^	0.126^b^	0.011	0.033^b^	NA	0.190^b^
Eo	0.013	0.020	0.000		0.069^b^	0.125^b^	0.021^a^	0.034^a^	NA	0.166^b^
Cam	0.141	0.173	0.160	0.122		0.025^a^	0.092^b^	0.114^b^	NA	0.175^b^
Red	0.195	0.246	0.210	0.209	0.041		0.117^b^	0.181^b^	NA	0.242^b^
Isl	0.038	0.070	0.020	0.039	0.167	0.200		0.01	NA	0.185^b^
Rio	0.038	0.060	0.061	0.062	0.203	0.301	0.020		NA	0.172^b^
Mur	NA	NA	NA	NA	NA	NA	NA	NA		NA
Del	0.219	0.300	0.334	0.294	0.300	0.393	0.336	0.306	NA	

NA, Not analyzed.

a Significant at *P* < 0.05.

b Significant after sequential Bonferroni correction.

Hierarchical analyses of molecular variance (Table 4) revealed that, when an Atlantic/Mediterranean subdivision was considered, a considerable percentage of variation was presented among groups (15.8%). But, this analysis also showed that the Atlantic group is heterogeneous (*F*_SC_ = 0.033, *P *<* *0.01). When considering the Mediterranean and three groups in the Atlantic (Cantabrian Sea/Rías Baixas/Gulf of Cadiz), or only these three groups, the percentage of variation within group was, in practical terms, zero (*F*_SC_ = −0.018). The percentage of variation among groups was significant irrespective of whether the Mediterranean sample was included or not (10.8% and 6.8%, respectively).

**Figure 2:**
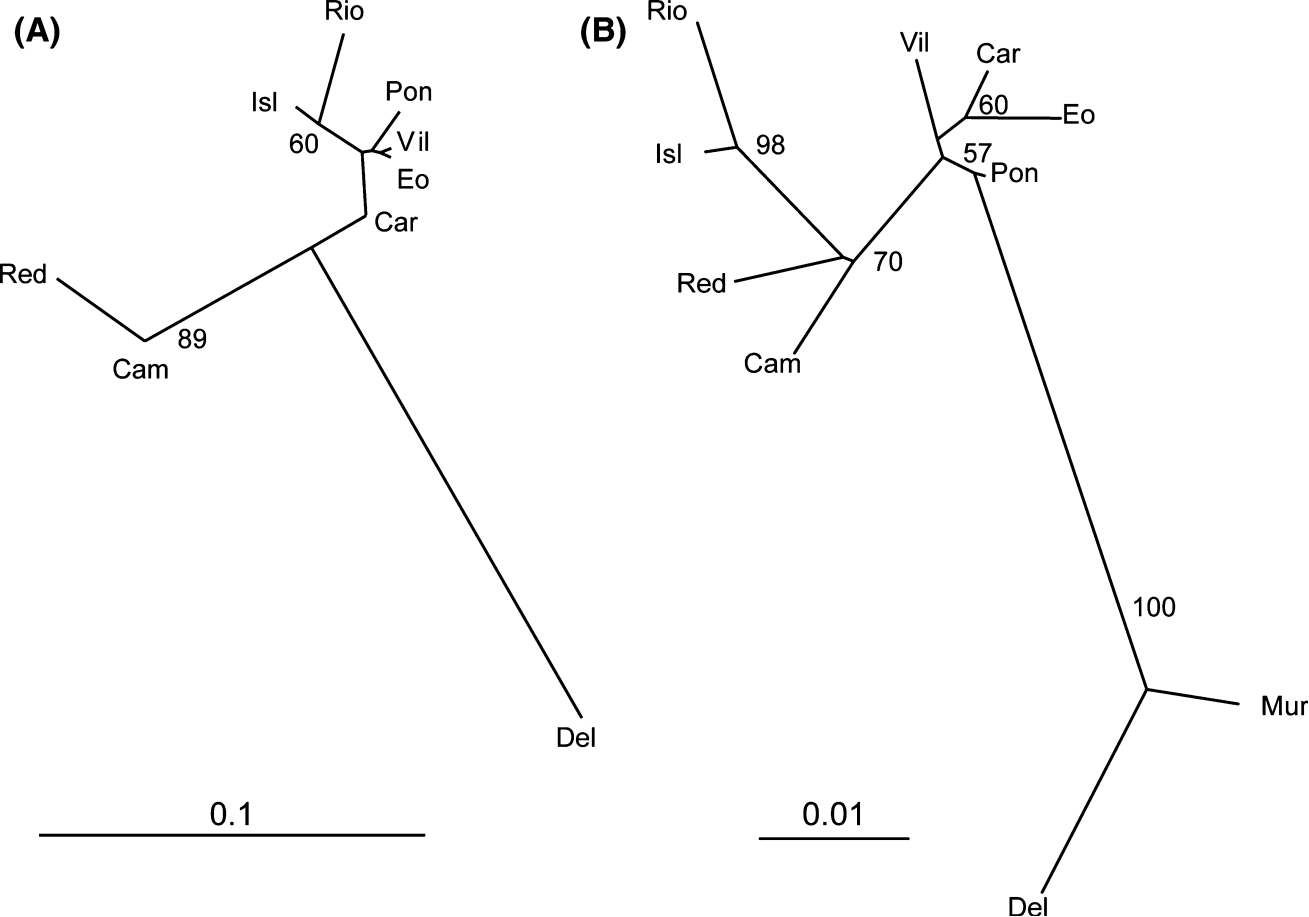
Neighbor‐joining tree based on genetic distances between samples. (A) Nei distances, computed from intronic data. (B) Reynolds's distances, calculated from microsatellite data. Numbers near nodes are bootstrap confidence values.

The neighbor‐joining tree based on Nei genetic distances (Fig. 2A) showed a polytomy with three divergent branches. These branches separated, respectively, the only Mediterranean population scored for intron variability (Del), the two populations from Rías Baixas, and the group formed by the populations from the Cantabrian coasts and the Gulf of Cadiz.

**Figure 3:**
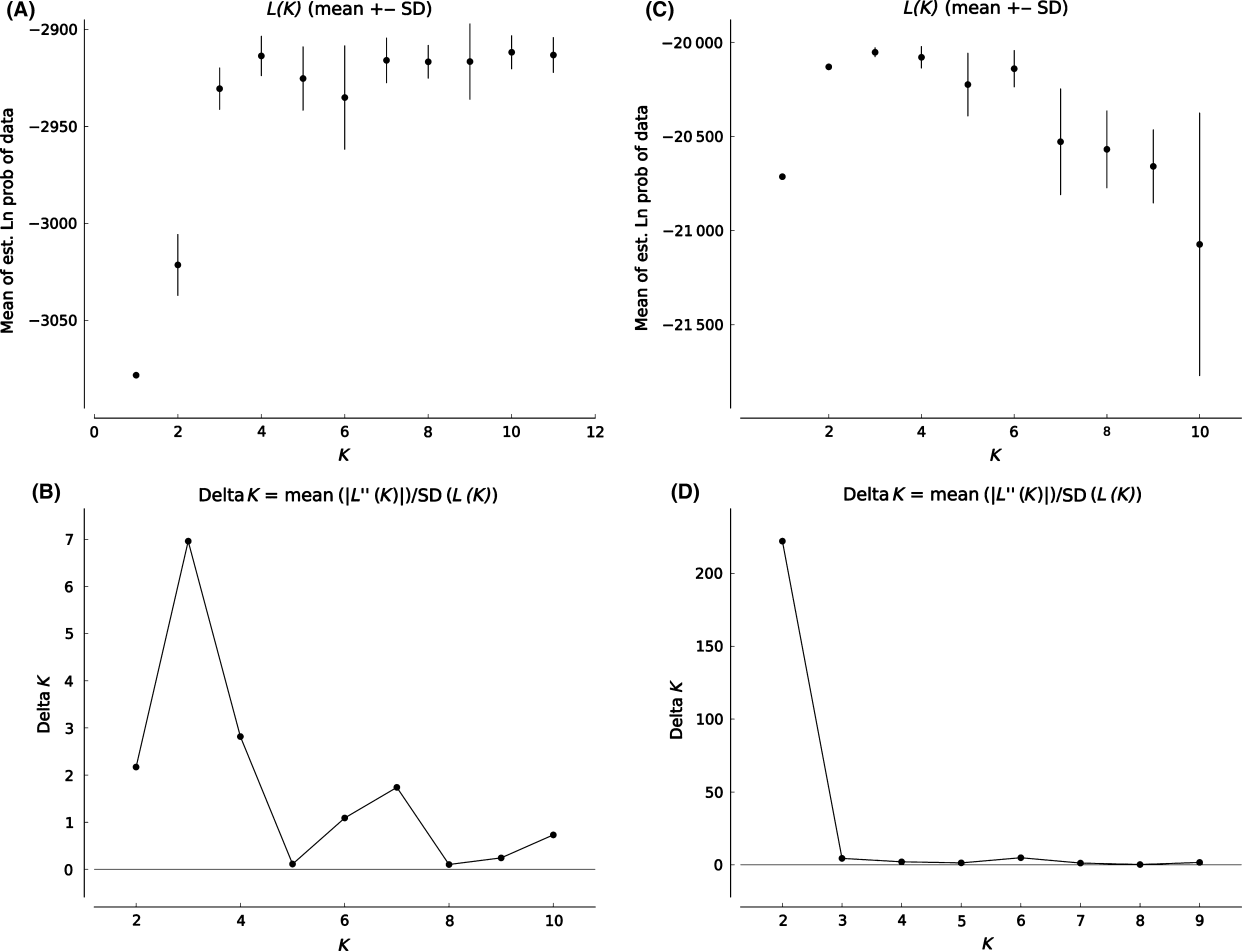
Bayesian analysis of genetic structure from intronic (A and B) and microsatellite (C and D) data. (A and C) Distribution of the Estimated log Likelihood of *K*,* L*(*K*). (B and D) Δ*K* as a function of *K*. For *L*(*K*) each point corresponds to the mean *L*(*K*) ± SD across 20 independent runs.

**Figure 4:**
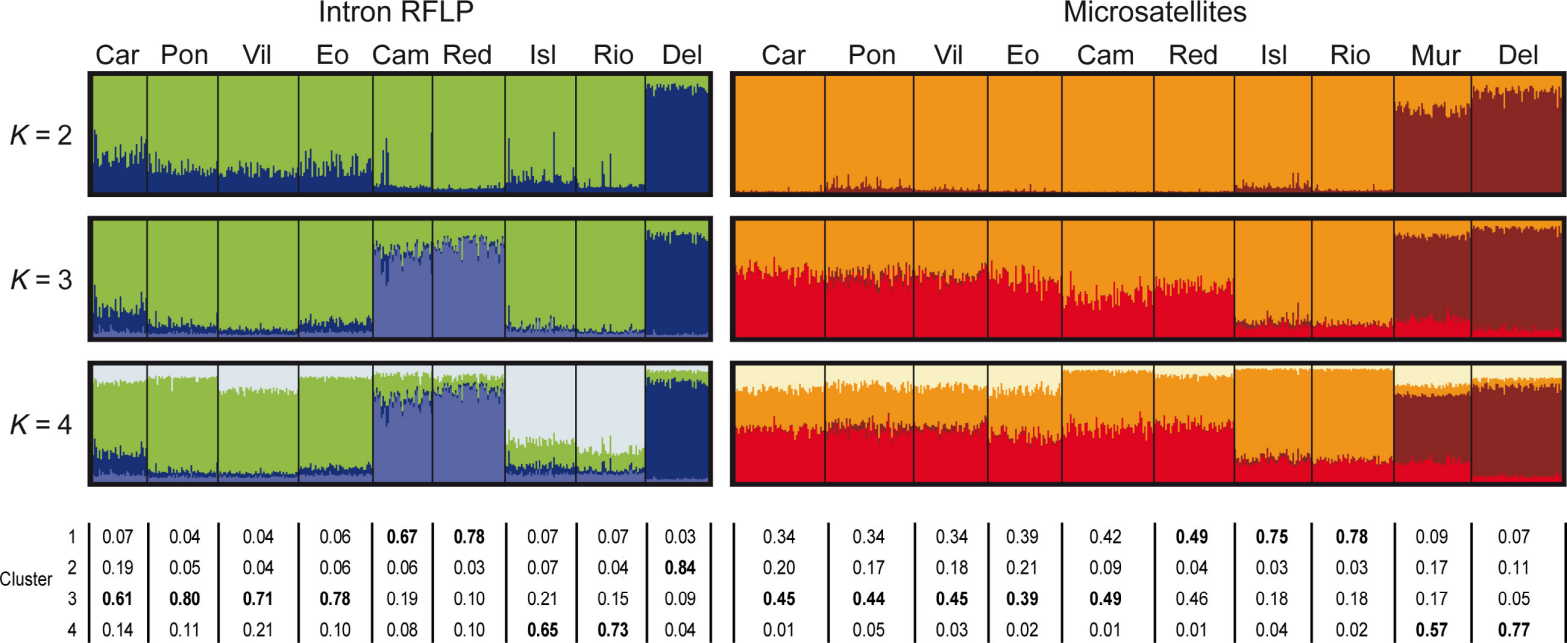
Graphical representation of the estimated membership coefficients for each individual obtained from the Bayesian clustering analysis of genetic structure for *K* = 2–4 computed from introns and microsatellites. Each individual is represented by a bar broken into *K* colored segments. Percentages of membership of each cluster to each population for *K* = 4 are given below the chart, with the most common cluster in each population shown in bold.

Figures 3 and 4 show the results of the Bayesian clustering analysis for three different number of clusters (*K*). Overall Obstruct *R* ^2^ values for *K* = 2–4 varied between 0.91 and 0.98 and were highly significant (*P *<* *0.001), indicating strong correlation between inferred ancestries and predefined populations and therefore strong population structure. The values of ln *P* (*X*|*K* Pritchard et al. (2000) and Δ*K* (Evanno et al. 2005) were highest for *K* = 3 (Fig. 3), but high values at *K* = 4 indicated additional structure in the Atlantic. A model with *K* = 2 showed two clusters that clearly divided samples belonging to the Mediterranean from the Atlantic samples, according to the differences in cluster frequencies. With *K* = 3, the populations from Rías Baixas were distinguished from the remaining Atlantic, and with *K* = 4, four geographic regions could be distinguished: Cantabrian Sea, Rías Baixas, Gulf of Cadiz, and Mediterranean (Fig. 4).

**Figure 5:**
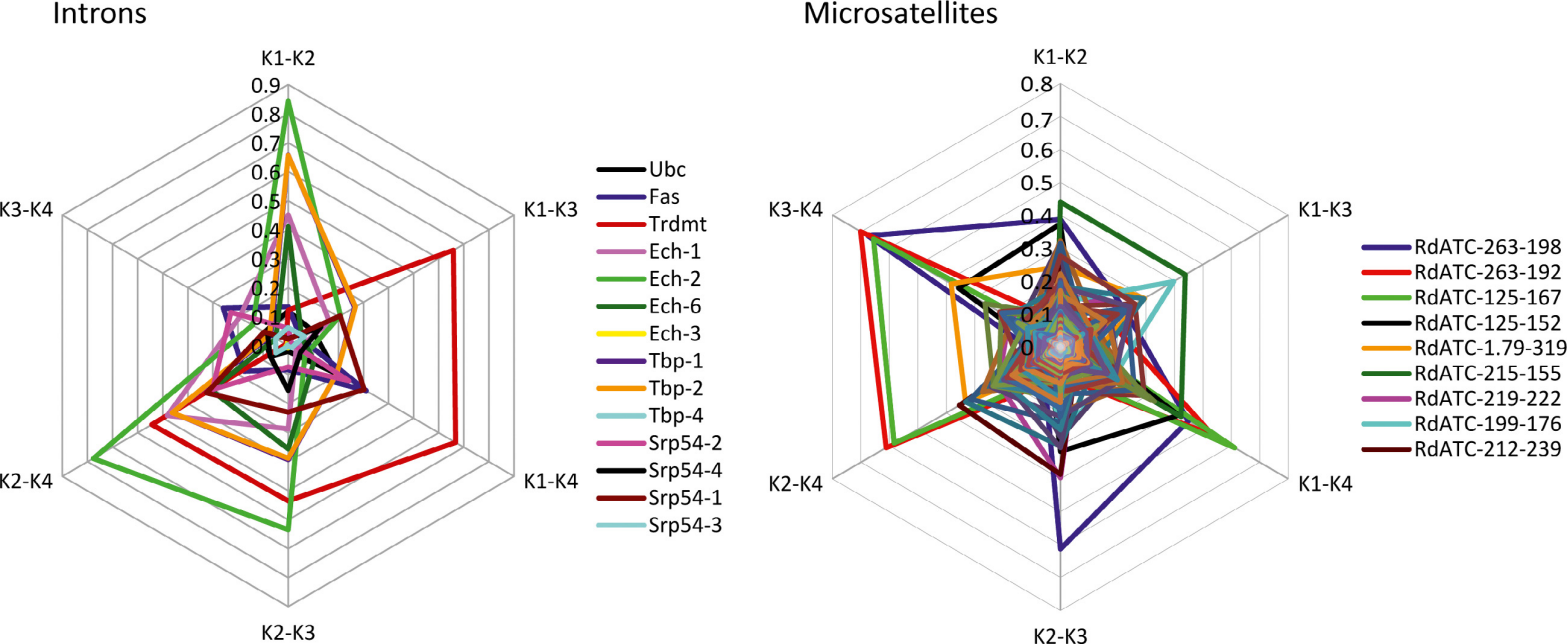
Allelic contributions to the differentiation of the clusters inferred by the Bayesian clustering analysis for *K* = 4, for introns and microsatellites. Each allele is represented with a color line. Intersections with axes show the differences in allele frequencies between clusters for each cluster pair. Biallelic intron markers are named by its locus name as both alleles contribute the same to differentiation of clusters. Due to the intense allele pattern superposition, only the nine microsatellite alleles showing the most conspicuous patterns have been named in the legend.

The contributions of the alleles of each intron marker to the differences among clusters are shown in the radar plot of Figure 5 for *K* = 4, which is the most complex structure. Several alleles at loci *Ech* and *Tbp*, especially *Ech*‐2 and *Tbp*‐2, displayed strong differences in frequency between cluster 2, the main cluster found in the Mediterranean samples, and the remaining clusters. On the other hand, the locus *Trdmt* showed the most important contribution to differentiate clusters 1 and 2 from clusters 3 and 4, and therefore to distinguish the groups of populations of Rias Baixas and Gulf of Cadiz, which are characterized by different frequencies of those two cluster groups between them and with respect to the Cantabrian and Mediterranean populations. Allele *Srp*54‐1 showed a similar pattern, although less marked.

#### Microsatellites

The analysis of genetic differentiation was carried out using 13 loci. Locus RdATC‐022, which showed linkage disequilibrium with RdATC‐199, was ignored. The global multilocus estimate for *F*_ST_ was significantly different from zero (*F*_ST_ = 0.029, *P *≤* *0.001; *F*_ST_’ = 0.088), indicating the existence of genetic differentiation among samples. The *F*_ST_ values per locus (Table 1) ranged from 0.012 (RdATC‐238) to 0.098 (RdATC‐263), showing all markers *P* values lower than 0.0001, with the exception of RdATC‐223 (*P *<* *0.023). Standardized values (*F*_ST_’) ranged from 0.013 to 0.261 (Table 1). When the *F*_ST_ statistic was calculated for Atlantic (*F*_ST_ = 0.013; *F*_ST_’ = 0.038) and Mediterranean (*F*_ST_ = 0.011; *F*_ST_’ = 0.063) regions separately, its value dropped by more than 50% but it was still significant (*P *<* *0.001).

Pairwise multilocus *F*_ST_ (Table 3) ranged from −0.001 (Pon‐Vil and Car‐Eo) to 0.075 (Rio‐Del), being significant after sequential Bonferroni correction 37 of 45 tests. Samples from the same region showed nonsignificant pairwise *F*_ST_ estimates with the exception of Mur and Del. Notably, pairwise *F*_ST_ values for comparisons involving a Mediterranean sample were, on average, four times higher, even for localities that are relatively close to the Mediterranean sea, as Isl and Rio.

**Table 3:** *F*_ST_ (above the diagonal) and *F*_ST_’ (below the diagonal) between pairs of localities of *Ruditapes decussatus*, estimated from microsatellite markers

	Car	Pon	Vil	Eo	Cam	Red	Isl	Rio	Mur	Del
Car		0.001	0.002	−0.001	0.016^b^	0.015^b^	0.026^b^	0.032^b^	0.041^b^	0.052^b^
Pon	0.002		−0.001	0.003	0.010^b^	0.012^b^	0.018^b^	0.029^b^	0.035^b^	0.044^b^
Vil	0.005	0.000		0.003	0.013^b^	0.014^b^	0.019^b^	0.030^b^	0.044^b^	0.050^b^
Eo	0.000	0.010	0.009		0.016^b^	0.015^b^	0.015^b^	0.021^b^	0.050^b^	0.057^b^
Cam	0.044	0.034	0.042	0.042		0.006^a^	0.012^b^	0.015^b^	0.052^b^	0.066^b^
Red	0.048	0.030	0.041	0.047	0.019		0.012^b^	0.014^b^	0.049^b^	0.064^b^
Isl	0.076	0.053	0.057	0.043	0.034	0.035		0.001	0.054^b^	0.063^b^
Rio	0.090	0.083	0.085	0.060	0.039	0.044	0.002		0.065^b^	0.075^b^
Mur	0.139	0.118	0.150	0.166	0.158	0.172	0.177	0.207		0.011^b^
Del	0.175	0.149	0.170	0.188	0.205	0.219	0.207	0.238	0.042	

a Significant at *P* < 0.05.

b Significant after sequential Bonferroni correction.

Different regional groups were tested using an AMOVA (Table 4). The highest percentage of variation among groups was observed when samples were grouped into two regions (Atlantic/Mediterranean) (% of variation among groups = 4.4; *F*_CT_ = 0.044). Nevertheless, this grouping also showed a significant within‐groups component (1.2%, *P *<* *0.001). When Atlantic samples were further subdivided (Cantabrian/Rías Baixas/Gulf of Cadiz), the among‐groups and within‐groups components dropped (1.5%, *P *<* *0.001, and 0.2%, *P *<* *0.001, respectively). *F*_CT_ and *F*_CT_’ in this case were 0.015 and 0.043, respectively.

**Table 4:** Partitioning of genetic variation with hierarchical *F*‐statistics in *Ruditapes decussatus*

Marker type	Subdivision levels^a^	*F*‐statistics			% of variation			Standardized *F*‐statistics		
		*F*_ST_	*F*_SC_	*F*_CT_	Among groups	Among populations (within groups)	Within Populations	*F*_ST_’	*F*_SC_’	*F*_CT_’
Introns	2	0.186^d^	0.033^d^	0.158	15.8	2.8	81.4	0.277	0.114	0.231
	3	0.051^d^	−0.018	0.068^b^	6.8	−1.6	94.9	0.127	0.032	0.115
	4	0.092^d^	−0.018	0.108^c^	10.8	−1.6	90.8	0.181	0.033	0.168
Microsatellites	2	0.057^d^	0.013^d^	0.044^d^	4.4	1.2	94.3	0.153	0.040	0.141
	3	0.018^d^	0.002^b^	0.015^d^	1.5	0.2	98.2	0.045	0.008	0.043
	4	0.035^d^	0.004^d^	0.031^d^	3.1	0.4	96.5	0.096	0.015	0.090

a Two levels: Atlantic versus Mediterranean Sea. Three levels: Cantabrian versus Rías Baixas versus Gulf of Cadiz. Four levels: Cantabrian Sea versus Rías Baixas versus Gulf of Cadiz versus Mediterranean Sea.

b *P *<* *0.05.

c *P *<* *0.01.

d *P *<* *0.001.

The neighbor‐joining tree (Fig. 2) clearly separated Mediterranean and non‐Mediterranean samples. Gulf of Cadiz samples clustered with Rías Baixas populations despite these samples being geographically closer to those from the Cantabrian Sea.

Figures 3 and 4 shows the results of the Bayesian clustering analysis for three values of *K*. Obstruct analysis showed high overall *R* ^2^ values (*R* ^2^ ≥ 0.97). The maximum value for the “estimated likelihood of *K*” was observed for *K* = 3, but *K* values of 2 and 4 showed similar values (Fig. 3). The highest Δ*K* value was obtained for *K* = 2 (Fig. 4), and with this *K* two differentiated population groups could be distinguished. Analyses with *K*3 and *K*4 suggest some differentiation of Gulf of Cadiz samples(Fig. 4).

The contribution of different alleles to clusters was studied for *K* = 4 (Fig. 5). Two alleles, RdATC263‐192 and RdATC125‐167, contributed greatly to the differentiation of cluster 4, the main cluster in the Mediterranean samples. Allele RdATC215‐155 had a high influence in differentiating cluster 1 from the others, and therefore in distinguishing Gulf of Cadiz populations.

### Test for isolation by distance

**Figure 6:**
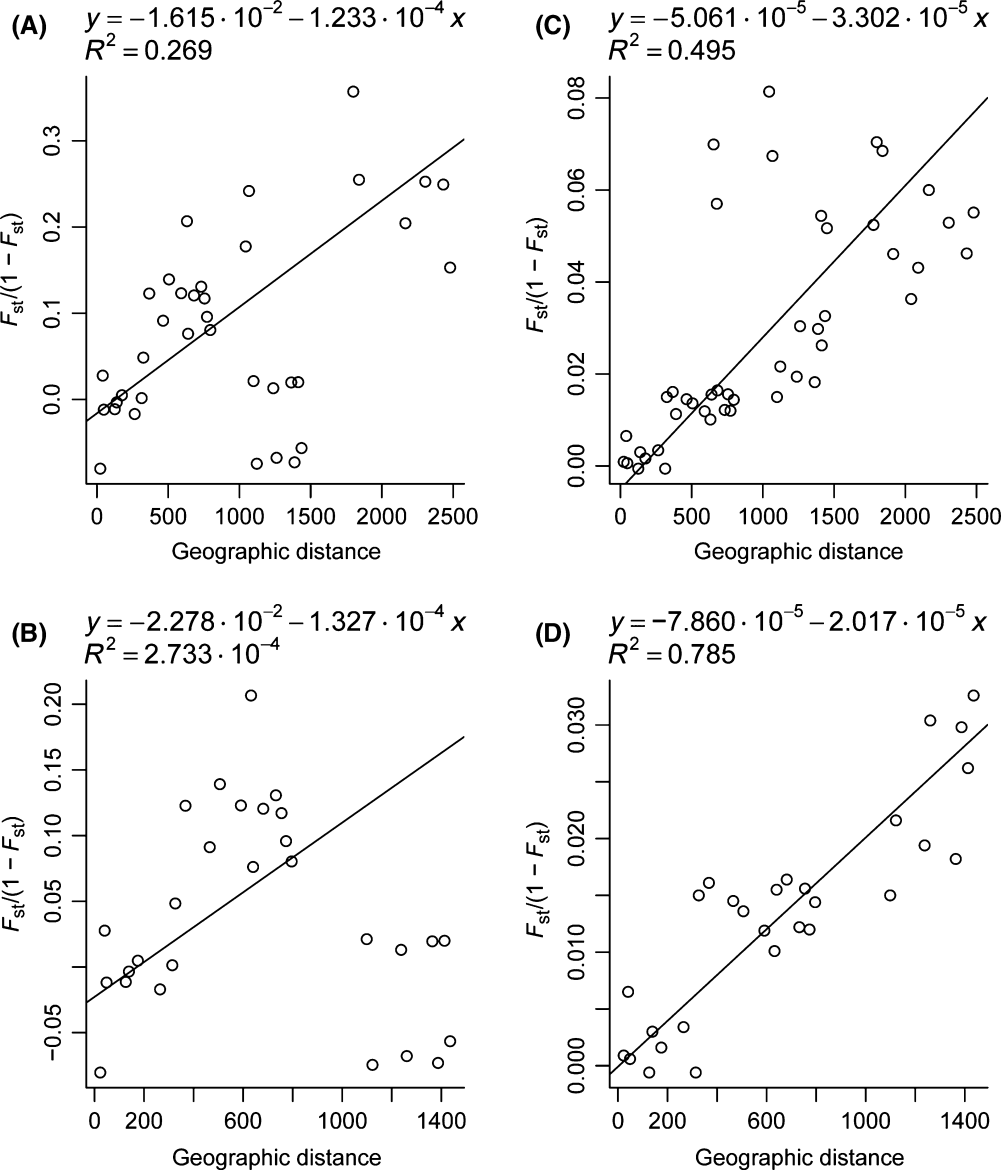
Tests for isolation by distance. (A) and (C) include all samples, and (B) and (D) Atlantic populations only. The charts on the left are based on intronic data and those in the right are based on microsatellite data.

No significant correlation was observed between geographic distance and the test statistic for an IBD model for intronic markers (*r *=* *0.518, *P *=* *0.075) (Fig. 6). Correlation was even lower when only the Atlantic populations were taken in account (*r *=* *0.017, *P *=* *0.395). However, for microsatellites, the Mantel test indicated that the degree of genetic differentiation increased significantly with distance (*r *=* *0.704, *P *=* *0.002) indicating support for an isolation by distance (IBD) model (Fig. 6). When the two Mediterranean samples were removed from the analysis, an IBD model continued to be supported for the remaining samples, showing even a higher correlation (*r *=* *0.886, *P *<* *0.001).

### Test for *F*_ST_ outliers

The test was carried out separately for introns and microsatellites because the different mutation rates that characterize the two types of markers could bias the result if they were pooled (Foll and Gaggiotti 2008). Results are given in Table 1. No intron was significant at the established false discovery rate of 5%, although SRP54 would be significant if a FDR = 10% were selected. As to microsatellites, four loci were significant at 5% FDR. These loci were RdATC‐1.34, RdATC‐1.79, RdATC‐177, and RdATC‐215. One more locus (RdATC‐238) was near the significance threshold. The *F*_ST_ for these loci were the lowest recorded. Values of alpha were negative in all significant cases. Results were similar when only Atlantic populations were analyzed.

## Discussion

We have analyzed the variability at 20 molecular markers (six intronic RFLP markers and 14 microsatellites) in 10 populations of *R. decussatus* distributed along the Spanish coasts. The results obtained from intron RFLPs both for intrapopulation genetic variability and interpopulation differentiation were very similar to those reported by Cordero et al. (2014) for other populations in the same regions. However, we found differences in some results produced by the two types of markers in several population genetic parameters, which resulted in a more complex picture than suspected from previous studies with allozymes and introns at the regional level.

The analysis of *F*_ST_ outliers for the two types of markers by the method of Foll and Gaggiotti (2008) showed that no introns could be considered as an outlier and therefore a potential target of natural selection. When applied to microsatellites, the test rendered four loci significant at 5% FDR. All the significant loci showed negative values of alpha. The test is known to produce a low amount of false positives for both positive and negative values (Foll and Gaggiotti 2008; Pérez‐Figueroa et al. 2010; Narum and Hess 2011; Gagnaire et al. 2015), so our results seem to not indicate important deviations from neutrality. This should be taken with caution given the small number of introns scored.

#### Genetic variability within populations

Measures of genetic variability (heterozygosity and allelic richness) were lower at introns than at microsatellites, a result that could be anticipated from the specific molecular nature of both types of markers. For introns, variation in *N*_*a*_ and *H*_*e*_ across populations was moderate, with no particular patterns of change along the sampled coasts. These results are in line with those reported by Cordero et al. (2014) for the Atlantic and West Mediterranean populations, and Borsa et al. (1994) in their allozyme study. However, microsatellites showed clearly higher values of *N*_*a*_ and *H*_*e*_ in Del and Mur, suggesting that the clams from West Mediterranean populations could harbor more genetic variability than those from the Atlantic populations. Examination of a larger number of clam populations from the West Mediterranean will be necessary to assess the generality of this observation. Launey et al. (2002) also reported higher variability at microsatellites in Mediterranean populations of the European flat oyster (*Ostrea edulis*) as compared to the Atlantic.

#### Genetic differentiation and gene flow

Genetic differentiation among populations, measured as *F*_ST_, was 0.029 for microsatellites and 0.088 for introns. These values are not directly comparable due to strong differences in the levels of variability and mutation rates for each type of marker (Hedrick 1999, 2005). The standardized *F*_ST_ measure (*F*_ST_’) proposed by Hedrick (2005), and extended by Meirmans (2006) to hierarchical *F*‐statistics, provides a mean to make a meaningful comparison (Tables 1, 4). Average values of *F*_ST_’ for introns (0.161 ± 0.050, ranging from 0.016 to 0.337) were higher than for microsatellites (0.105 ± 0.018, ranging from 0.013 to 0.261), but the difference of mean *F*_ST_’ between introns and microsatellites was not statistically significant.

The pattern of population differentiation differed between markers. While microsatellites fitted an isolation by distance model in the total area and in the Atlantic, introns did not. A potential interpretation of these results is that introns have lower power than microsatellites to detect an IBD pattern due to the low number of alleles per locus and the lower number of loci scored. Alternatively, the IBD detected at microsatellites could be a spurious result of the subdivided genetic structure of clam populations (Meirmans 2012).

Both types of markers showed that the main source of genetic differentiation among clam populations was the Atlantic–Mediterranean divide, and confirm a similar observation by Cordero et al. (2014) recorded with introns alone. These results support the view held by those authors that neutral mechanisms (*i.e*., historical subdivision and gene flow restrictions) are responsible for the genetic differentiation of clam populations from the two marine basins. This differentiation is usually explained in the framework of sea level changes caused by Pleistocene glaciations (Patarnello et al. 2007; Cordero et al. 2014). Our results also suggest that low genetic differentiation between the Atlantic and West Mediterranean clam populations recorded by Borsa et al. (1994) using allozyme markers could reflect the action of natural selection on proteins.

Atlantic populations showed also significant genetic differentiation. Here, significant *F*_ST_ values of 0.051 and 0.013 were found for introns and microsatellites, respectively The value obtained with microsatellites is of similar magnitude to the values reported in other bivalve species, such as the European oyster (*Ostrea edulis*) (0.000–0.022; Launey et al. 2002) or the Mediterranean mussel (*Mytilus galloprovincialis*) (average of all pairwise *F*_ST_ = 0.02; Diz and Presa 2008). Using the well‐known relationship between *F*_ST_ and the product of the effective population size and the migration rate (*N*_*e*_ *m*), which is the effective number of migrants (Slatkin 1985), we obtained estimates of *N*_*e*_ *m* of 4.6 and 19, respectively. Thus, genetic differentiation occurs but with high connectivity and gene flow along the Atlantic coast. Our analysis with hierarchical *F*‐statistics indicates that there is also significant regional substructure within the Atlantic. With several methods (pairwise *F*_ST_, Bayesian analysis of the genetic structure, neighbor‐joining trees), we have distinguished three regions that are genetically different: Cantabrian Sea, Rias Baixas, and the Gulf of Cadiz. It remains to be seen whether the genetic differentiation detected reflects a genetic discontinuity between the three Atlantic regions investigated here, or whether there is a continuous genetic variation along the Atlantic coasts. This will require a more regularly spaced population sampling as well as including the coasts of Portugal in it.

It is interesting to note that the group formed by the two populations from Rias Baixas was distinguished only by introns (Figs. 2, 4). The Bayesian analysis of genetic structure showed an increased frequency of cluster 1 and a decrease of cluster 3 of intronic markers in these populations. These clusters are mostly differentiated by the locus *Trdmt* and, to a lower extent, by locus *Srp54* (Fig. 5). These two loci have in common that they showed a very specific pattern of geographic variation in the study of Cordero et al. (2014), which consisted in significant population genetic differentiation among populations (*F*_ST_) across the whole area of study and a lack of significant differentiation among the three clam races (*F*_CT_). This pattern contrasted with the pattern presented by the remaining 4 loci, which was characterized by significant, usually high, differentiation between races. Cordero et al. (2014) argued that these contrasting patterns of variation among loci could be explained by the existence of endogenous barriers to gene flow among races affecting the loci with high *F*_CT_ values, but inactive in the genome regions surrounding *Trdmt* and *Srp54*. These observations suggest that the same factors that caused the lack of differentiation among races at *Trdmt* and *Srp54* in the study of Cordero et al. (2014) could be also responsible for the differentiation of the Rias Baixas populations observed in the present study. Moreover, *Trdmt* was the locus with the highest *F*_SC_ value in the study of Cordero et al. (2014) and showed the highest *F*_ST_ in this study. High *F*_ST_ values are often associated with loci experiencing directional or diversifying selection. Although the test of Foll and Gaggiotti (2008) did not detect the *F*_ST_ value of *Trdmt* as an outlier, this result should be taken with caution given the short number of introns scored. The possibility that selection acting on *Trdmt,* or on linked regions, is involved in the differentiation of Rias Baixas populations should be examined in more detail in future studies.

Significance tests (Tables 2, 3) detected no genetic differentiation among the populations of the Cantabrian Sea and the Gulf of Cadiz for microsatellites and introns. However, genetic differentiation among populations was detected within the Rias Baixas and the West Mediterranean subregions. The two Mediterranean populations were differentiated for both marker types. These populations come from semi‐enclosed habitats, and therefore, the significant differentiation exhibited is not unexpected. The differences between the two populations from Rias Baixas could be the result of a relative isolation of the estuaries, in spite of being open to the ocean, due to their specific patterns of current circulation.

The introduction of the Manila clam and the supplementation programs at some localities are two facts that might have affected the genetics of wild populations of grooved carpet‐shell clam in recent decades. It is clear that some hybridization with Manila clam has taken place at Ria de Vigo and in some localities in the Cantabrian coast (Hurtado et al. 2011; Habtemariam et al. 2015). We think that this hybridization has not affected sensibly the results of our study. One reason is that the level of hybridization has been probably very low, ranging from 1% to 3% (Hurtado et al. 2011; Habtemariam et al. 2015). In Ría de Vigo, hybridization has not been found again after the initial record in 2006 (J.J Pasantes, University of Vigo, personal communication). These observations suggest that hybridization is a very occasional phenomenon. On the other hand, the markers used here were tested in Manila clam and either they simply did not amplify at all in that species, or, in the case of some introns, produced fragments of very different size. Therefore, substantial hybridization and introgression would have led to an increase of homozygosity and of null homozygotes at all loci, or to clearly different genotypes at some introns. We have observed no strange intronic RFLP patterns, and only a few deviations from Hardy–Weinberg equilibrium at some scattered loci, which supports that introgression is not affecting our results.

Supplementation could lead to a decrease or an increase of genetic differentiation of supplemented populations, depending on a number of circumstances. In all cases, supplementation levels should be very high to produce an appreciable shift in overall gene frequencies in the population. This is not a realistic situation, since clam captures are limited by clam size and catch size in order to allow the persistence of the populations, and therefore, wild noncaptured clams should greatly outnumber the spat released for restocking. In addition, recurrent supplementation in different years with spat from different batches of breeders would erase previous patterns. Therefore, it is improbable that supplementation would have led to genetic differentiation patterns observed.

#### Managing the genetic resources of the grooved carpet‐shell clam

Our results have rendered the most complete characterization to date of wild exploited populations of *R. decussatus*. Here, we have shown that genetic homogeneity is a characteristic feature of all the populations distributed along the Spanish coast of the Cantabrian Sea. The same applies to the Gulf of Cadiz. These data are useful for designing population managing strategies. Specifically, it seems that whole regions rather than individual localities should be the units of management in this species in these two areas. This represents an advantage because larger base populations facilitate the conservation of higher levels of genetic variability and the achievement of low inbreeding in supplemented populations in recurrent restocking programs. The Mediterranean populations are not exploited currently, but the preservation of its genetic distinctness and high genetic variability is important because they represent a genetic reservoir that could be useful for future breeding plans in this species. The situation regarding Rias Baixas is not so clear as the two samples analyzed here showed relatively higher *F*_ST_ values than neighbor populations in other regions, and these were statistically significant for both marker types. This suggests that each different estuary (*ria*) should be considered a separate management unit. However, this will not be clearly established until more populations within each *ria*, other *rias*, and nearby areas in Northwest Spain are characterized in more detail. This is an urgent task as they contribute the majority of the Spanish clam production.

## Conflict of Interest

None declared.

## Appendix 1 Allele frequencies, sample sizes (N), number of alleles (*N*a), expected (*H*e) and observed (*H*o) heterozygosities, and deviations from Hardy–Weinberg equilibrium (as measured by *F*is)in ten populations of *R. decussatus*. Locality abbreviations as in Fig. 1.

**Table:**

Marker	Allele		Population									
			Car	Pon	Vil	Eo	Cam	Red	Isl	Rio	Del	All
*Ech*	A		0.471	0.409	0.400	0.304	0.378	0.618	0.489	0.543	0.128	
	B		0.324	0.205	0.210	0.250	0.081	0.074	0.170	0.086	0.782	
	C		0.000	0.000	0.020	0.000	0.000	0.000	0.000	0.014	0.026	
	Null		0.206	0.386	0.370	0.446	0.541	0.309	0.341	0.357	0.064	
		*N*	34	44	50	46	37	34	44	35	39	
		*N*a	3	3	4	3	3	3	3	4	4	4
		*H*e	0.641	0.649	0.665	0.653	0.566	0.525	0.623	0.578	0.372	
		*H*o	0.559	0.614	0.640	0.652	0.514	0.412	0.591	0.457	0.359	
		*F*is	0.572^a^	0.586^b^	0.569^b^	0.626^b^	0.871^b^	0.525^a^	0.605^b^	0.584^a^	0.203	0.569
*Fas*	A		0.912	0.929	0.878	0.933	0.972	0.942	0.686	0.762	0.851	
	B		0.088	0.071	0.122	0.067	0.028	0.058	0.314	0.238	0.149	
		*N*	34	42	49	45	36	43	43	42	37	
		*N*a	2	2	2	2	2	2	2	2	2	2
		*H*e	0.163	0.134	0.217	0.126	0.055	0.111	0.436	0.367	0.257	
		*H*o	0.177	0.095	0.204	0.133	0.000	0.116	0.535	0.429	0.297	
		*F*is	−0.082	0.293	0.061	−0.060	1.000^a^	−0.050	−0.231	−0.170	−0.161	−0.066
*Srp54*	A		0.242	0.438	0.216	0.228	0.413	0.450	0.226	0.216	0.466	
	B		0.500	0.417	0.534	0.565	0.478	0.483	0.643	0.689	0.431	
	C		0.081	0.021	0.057	0.054	0.022	0.000	0.036	0.000	0.052	
	D		0.177	0.125	0.193	0.152	0.087	0.067	0.095	0.095	0.052	
		*N*	31	24	44	46	23	30	42	37	29	
		*N*a	4	4	4	4	4	3	4	3	4	4
		*H*e	0.664	0.632	0.635	0.609	0.606	0.569	0.532	0.476	0.603	
		*H*o	0.774	0.750	0.659	0.544	0.478	0.300	0.500	0.378	0.586	
		*F*is	−0.169	−0.191^a^	−0.039	0.109	0.214^a^	0.477^a^	0.060^b^	0.207^b^	0.028	0.068
*Tbp*	A		0.426	0.464	0.337	0.359	0.327	0.156	0.341	0.625	0.729	
	B		0.574	0.536	0.663	0.630	0.673	0.844	0.659	0.375	0.271	
	C		0.000	0.000	0.000	0.011	0.000	0.000	0.000	0.000	0.000	
		*N*	27	42	49	46	26	45	41	4	35	
		*N*a	2	2	2	3 (1)	2	2	2	2	2	3 (1)
		*H*e	0.498	0.503	0.451	0.479	0.449	0.266	0.455	0.536	0.401	
		*H*o	0.482	0.548	0.429	0.435	0.423	0.267	0.342	0.250	0.257	
		*F*is	0.034	−0.089	0.051	0.093	0.058	−0.004	0.252	0.571	0.362	0.097
*Trdmt*	A		0.667	0.821	0.780	0.722	0.294	0.267	0.705	0.731	0.342	
	B		0.333	0.179	0.220	0.278	0.706	0.733	0.295	0.269	0.658	
		*N*	30	42	50	45	34	45	44	39	38	
		*N*a	2	2	2	2	2	2	2	2	2	2
		*H*e	0.452	0.297	0.347	0.406	0.421	0.396	0.421	0.399	0.456	
		*H*o	0.400	0.262	0.360	0.422	0.412	0.222	0.364	0.436	0.421	
		*F*is	0.117	0.119	−0.039	−0.041	0.023	0.441^a^	0.138	−0.095	0.078	0.085
*Ubc*	A		0.643	0.756	0.771	0.714	0.743	0.856	0.713	0.631	0.710	
	B		0.357	0.244	0.229	0.286	0.257	0.144	0.287	0.369	0.290	
		*N*	21	43	48	42	35	45	40	42	31	
		*N*a	2	2	2	2	2	2	2	2	2	2
		*H*e	0.470	0.373	0.357	0.413	0.388	0.250	0.415	0.471	0.419	
		*H*o	0.524	0.302	0.292	0.333	0.457	0.244	0.525	0.452	0.323	
		*F*is	−0.117	0.192	0.185	0.195	−0.183	0.022	−0.270	0.041	0.233	0.042
All		*N*a	2.500	2.500	2.667	2.667	2.500	2.333	2.500	2.500	2.667	
		*H*e	0.482	0.432	0.445	0.448	0.414	0.353	0.480	0.471	0.418	
		*H*o	0.486	0.429	0.431	0.420	0.381	0.260	0.476	0.400	0.374	

a Significant departures from Hardy–Weinberg equilibrium at *P* < 0.05.

b Significant departures from Hardy–Weinberg equilibrium after sequential Bonferroni correction.

## Appendix 2 Allelic frequencies and variability parameters at microsatellite loci in ten populations of *R. decussatus*. *N*: number of individuals; *Na*: observed number of alleles, with the number of private alleles in parentheses; *Rs*: allelic richness based on a minimum sample size of 41 individuals; *Ho*: observed heterozygosity; *He*: expected heterozygosity; *F*_*IS*_: inbreeding coefficient. Locality abbreviations as in Fig. 1.

**Table:**

Marker	Allele		Population										
			Car	Pon	Vil	Eo	Cam	Red	Isl	Rio	Mur	Del	All
RdATC‐022													
	131		0.000	0.000	0.000	0.011	0.000	0.010	0.000	0.000	0.031	0.036	
	134		0.000	0.000	0.054	0.022	0.000	0.000	0.000	0.000	0.000	0.027	
	137		0.205	0.173	0.087	0.109	0.237	0.310	0.146	0.150	0.333	0.255	
	140		0.027	0.036	0.065	0.054	0.035	0.060	0.010	0.030	0.010	0.009	
	143		0.277	0.309	0.337	0.326	0.263	0.310	0.479	0.520	0.354	0.427	
	146		0.295	0.309	0.348	0.315	0.395	0.230	0.208	0.210	0.240	0.209	
	149		0.152	0.082	0.065	0.098	0.061	0.050	0.042	0.060	0.000	0.018	
	152		0.018	0.082	0.044	0.054	0.009	0.020	0.083	0.010	0.021	0.018	
	155		0.027	0.009	0.000	0.011	0.000	0.010	0.010	0.020	0.010	0.000	
	158		0.000	0.000	0.000	0.000	0.000	0.000	0.021	0.000	0.000	0.000	
		*N*	56	55	46	46	57	50	48	50	48	55	
		*Na*	7	7	7	9	6	8	8 (1)	7	7	8	10 (1)
		*Rs*	6.894	6.742	7.000	8.772	5.714	7.609	7.688	6.784	6.686	7.601	7.716
		*He*	0.777	0.771	0.753	0.775	0.720	0.756	0.704	0.665	0.712	0.713	
		*Ho*	0.768	0.782	0.739	0.652	0.684	0.600	0.792	0.600	0.583	0.418	
		*Fis*	0.011	−0.014	0.018	0.160^a^	0.050	0.208	−0.127	0.098	0.182^a^	0.415^b^	0.100
RdATC‐1.34	229		0.009	0.000	0.000	0.000	0.009	0.000	0.000	0.000	0.000	0.000	
	232		0.000	0.046	0.046	0.120	0.009	0.060	0.042	0.029	0.080	0.146	
	235		0.098	0.091	0.068	0.076	0.053	0.100	0.063	0.059	0.057	0.036	
	238		0.161	0.173	0.205	0.174	0.132	0.120	0.260	0.137	0.034	0.000	
	241		0.080	0.055	0.068	0.044	0.035	0.040	0.042	0.108	0.011	0.018	
	244		0.223	0.273	0.307	0.348	0.360	0.260	0.344	0.245	0.091	0.109	
	247		0.125	0.091	0.080	0.076	0.132	0.090	0.115	0.167	0.341	0.364	
	250		0.223	0.218	0.159	0.130	0.219	0.250	0.115	0.226	0.284	0.264	
	253		0.071	0.036	0.068	0.033	0.044	0.060	0.021	0.010	0.080	0.064	
	256		0.009	0.018	0.000	0.000	0.009	0.010	0.000	0.020	0.000	0.000	
	259		0.000	0.000	0.000	0.000	0.000	0.000	0.000	0.000	0.023	0.000	
	262		0.000	0.000	0.000	0.000	0.000	0.010	0.000	0.000	0.000	0.000	
		*N*	56	55	44	46	57	50	48	51	44	55	
		*Na*	9	9	8	8	10	10 (1)	8	9	9 (1)	7	12 (2)
		*Rs*	8.464	8.932	8	7.999	9.151	9.639	7.979	8.76	8.928	6.933	8.804
		*He*	0.845	0.833	0.826	0.812	0.789	0.837	0.788	0.834	0.786	0.767	
		*Ho*	0.821	0.800	0.864	0.652	0.702	0.800	0.688	0.902	0.636	0.709	
		*Fis*	0.028	0.039	−0.046	0.198	0.111	0.044	0.129	−0.082	0.192	0.076^a^	0.066
RdATC‐1.79	293		0.000	0.000	0.000	0.000	0.000	0.000	0.000	0.000	0.011	0.000	
	297		0.000	0.000	0.000	0.000	0.000	0.000	0.000	0.000	0.011	0.000	
	301		0.009	0.000	0.000	0.000	0.000	0.000	0.000	0.000	0.011	0.000	
	304		0.000	0.000	0.000	0.000	0.000	0.000	0.010	0.010	0.011	0.000	
	307		0.036	0.027	0.033	0.022	0.035	0.031	0.010	0.020	0.046	0.009	
	310		0.027	0.000	0.011	0.000	0.026	0.000	0.031	0.039	0.000	0.009	
	313		0.018	0.036	0.011	0.033	0.026	0.020	0.063	0.049	0.159	0.194	
	316		0.134	0.146	0.120	0.174	0.184	0.102	0.156	0.157	0.080	0.278	
	319		0.321	0.400	0.370	0.250	0.290	0.306	0.156	0.167	0.159	0.204	
	322		0.152	0.136	0.217	0.207	0.167	0.225	0.135	0.186	0.193	0.093	
	325		0.063	0.073	0.054	0.054	0.070	0.071	0.115	0.039	0.114	0.056	
	328		0.107	0.073	0.065	0.098	0.114	0.133	0.146	0.167	0.068	0.056	
	331		0.071	0.046	0.044	0.130	0.035	0.061	0.063	0.088	0.068	0.037	
	334		0.054	0.036	0.033	0.011	0.018	0.020	0.094	0.059	0.000	0.009	
	337		0.000	0.000	0.033	0.022	0.035	0.010	0.010	0.010	0.068	0.019	
	340		0.009	0.018	0.000	0.000	0.000	0.010	0.000	0.000	0.000	0.009	
	343		0.000	0.009	0.011	0.000	0.000	0.010	0.000	0.010	0.000	0.028	
	352		0.000	0.000	0.000	0.000	0.000	0.000	0.010	0.000	0.000	0.000	
		*N*	56	55	46	46	57	49	48	51	44	54	
		*Na*	12	11	12	10	11	12	13 (1)	13	13 (2)	13	18 (3)
		*Rs*	11.372	10.659	11.671	9.869	10.866	11.456	12.414	12.372	12.727	11.965	11.792
		*He*	0.837	0.791	0.798	0.842	0.839	0.825	0.890	0.876	0.886	0.834	
		*Ho*	0.786	0.691	0.913	0.826	0.860	0.857	0.896	0.941	0.841	0.833	
		*Fis*	0.062	0.128	−0.146	0.019	−0.025	−0.039	−0.007^a^	−0.075	0.052	0.000	−0.001
RdATC‐125	146		0.071	0.093	0.065	0.087	0.123	0.090	0.229	0.200	0.188	0.107	
	149		0.000	0.009	0.011	0.000	0.000	0.000	0.010	0.010	0.031	0.054	
	152		0.446	0.472	0.522	0.446	0.518	0.540	0.552	0.580	0.250	0.304	
	155		0.098	0.093	0.087	0.098	0.097	0.150	0.146	0.110	0.146	0.063	
	158		0.009	0.000	0.022	0.000	0.018	0.000	0.000	0.010	0.115	0.188	
	161		0.018	0.046	0.022	0.054	0.044	0.020	0.031	0.010	0.083	0.027	
	164		0.009	0.028	0.033	0.000	0.044	0.000	0.000	0.010	0.052	0.045	
	167		0.321	0.250	0.196	0.283	0.088	0.170	0.021	0.060	0.073	0.161	
	170		0.027	0.009	0.022	0.033	0.070	0.020	0.010	0.010	0.063	0.045	
	173		0.000	0.000	0.000	0.000	0.000	0.010	0.000	0.000	0.000	0.000	
	176		0.000	0.000	0.022	0.000	0.000	0.000	0.000	0.000	0.000	0.009	
		*N*	56	54	46	46	57	50	48	50	48	56	
		*Na*	8	8	10	6	8	7 (1)	7	9	9	10	11 (1)
		*Rs*	7.376	7.505	9.847	5.999	7.92	6.758	6.686	8.1	8.997	9.712	8.673
		*He*	0.688	0.701	0.682	0.708	0.697	0.655	0.626	0.614	0.857	0.831	
		*Ho*	0.625	0.759	0.609	0.783	0.684	0.640	0.646	0.660	0.833	0.875	
		*Fis*	0.092	−0.084	0.109^a^	−0.107	0.019	0.022	−0.032	−0.077	0.028	−0.053	−0.008
RdATC‐177	219		0.009	0.000	0.000	0.000	0.000	0.000	0.000	0.000	0.000	0.000	
	225		0.009	0.082	0.054	0.011	0.018	0.010	0.094	0.059	0.146	0.143	
	228		0.000	0.009	0.000	0.000	0.000	0.010	0.021	0.029	0.037	0.116	
	231		0.018	0.009	0.011	0.011	0.000	0.000	0.031	0.000	0.073	0.232	
	234		0.009	0.018	0.033	0.034	0.009	0.010	0.031	0.029	0.024	0.071	
	237		0.080	0.073	0.076	0.068	0.070	0.070	0.167	0.137	0.098	0.196	
	240		0.071	0.046	0.098	0.114	0.070	0.070	0.135	0.069	0.049	0.045	
	243		0.161	0.200	0.207	0.182	0.149	0.160	0.115	0.157	0.110	0.027	
	246		0.161	0.173	0.174	0.114	0.263	0.170	0.156	0.147	0.061	0.036	
	249		0.205	0.209	0.141	0.182	0.167	0.240	0.115	0.118	0.195	0.027	
	252		0.179	0.109	0.163	0.193	0.167	0.140	0.063	0.167	0.146	0.071	
	255		0.063	0.046	0.033	0.057	0.053	0.070	0.031	0.049	0.061	0.018	
	258		0.036	0.027	0.011	0.011	0.009	0.040	0.031	0.029	0.000	0.009	
	261		0.000	0.000	0.000	0.011	0.026	0.010	0.010	0.010	0.000	0.009	
	267		0.000	0.000	0.000	0.011	0.000	0.000	0.000	0.000	0.000	0.000	
		*N*	56	55	46	44	57	50	48	51	41	56	
		*Na*	12	12 (1)	11	13 (1)	11	12	13	12	11	13	15 (2)
		*Rs*	11.122	11.411	10.781	12.659	10.341	11.279	12.824	11.784	11	12.353	12.202
		*He*	0.865	0.865	0.869	0.871	0.847	0.860	0.895	0.889	0.891	0.866	
		*Ho*	0.732	0.836	0.870	0.841	0.860	0.880	0.771	0.765	0.707	0.839	
		*Fis*	0.154^a^	0.033	0.000	0.035	−0.016	−0.023	0.140	0.141	0.208^a^	0.031	0.069
RdATC‐185	132		0.000	0.000	0.000	0.011	0.009	0.000	0.000	0.000	0.000	0.000	
	138		0.018	0.000	0.011	0.033	0.009	0.020	0.021	0.029	0.010	0.009	
	141		0.809	0.877	0.826	0.837	0.864	0.704	0.819	0.745	0.698	0.652	
	144		0.036	0.009	0.011	0.011	0.018	0.051	0.075	0.039	0.083	0.116	
	147		0.109	0.094	0.152	0.087	0.091	0.225	0.075	0.157	0.188	0.223	
	150		0.009	0.009	0.000	0.011	0.000	0.000	0.011	0.000	0.010	0.000	
	153		0.018	0.000	0.000	0.011	0.009	0.000	0.000	0.000	0.000	0.000	
	156		0.000	0.009	0.000	0.000	0.000	0.000	0.000	0.029	0.000	0.000	
	159		0.000	0.000	0.000	0.000	0.000	0.000	0.000	0.000	0.010	0.000	
		*N*	55	53	46	46	55	49	47	51	48	56	
		*Na*	6	5	4	7	6	4	5	5	6 (1)	4	9 (1)
		*Rs*	5.616	4.321	3.783	6.564	5.173	3.975	4.857	4.986	5.562	3.732	4.881
		*He*	0.334	0.223	0.297	0.294	0.248	0.456	0.321	0.421	0.475	0.516	
		*Ho*	0.291	0.245	0.348	0.304	0.218	0.449	0.340	0.490	0.521	0.500	
		*Fis*	0.131^a^	−0.100	−0.172	−0.037	0.120	0.014	−0.062	−0.166	−0.097	0.032	−0.030
RdATC‐199	164		0.000	0.000	0.000	0.000	0.000	0.010	0.000	0.000	0.021	0.028	
	167		0.000	0.000	0.000	0.022	0.000	0.000	0.000	0.000	0.000	0.019	
	170		0.209	0.176	0.163	0.109	0.232	0.276	0.163	0.128	0.313	0.185	
	173		0.027	0.019	0.044	0.044	0.036	0.061	0.011	0.029	0.010	0.009	
	176		0.273	0.296	0.348	0.380	0.259	0.306	0.467	0.510	0.240	0.306	
	179		0.291	0.324	0.337	0.261	0.411	0.235	0.185	0.216	0.271	0.259	
	182		0.164	0.148	0.098	0.152	0.063	0.082	0.130	0.088	0.010	0.028	
	185		0.018	0.009	0.000	0.000	0.000	0.010	0.022	0.020	0.010	0.000	
	188		0.018	0.028	0.011	0.033	0.000	0.020	0.022	0.010	0.125	0.167	
		*N*	55	54	46	46	56	49	46	51	48	54	
		*Na*	7	7	6	7	5	8	7	7	8	8	9
		*Rs*	6.859	6.69	5.891	6.988	4.996	7.648	6.87	6.76	7.543	7.678	7.117
		*He*	0.776	0.760	0.735	0.757	0.712	0.772	0.711	0.675	0.763	0.783	
		*Ho*	0.800	0.815	0.804	0.717	0.679	0.633	0.783	0.667	0.792	0.852	
		*Fis*	−0.031	−0.073	−0.095	0.053	0.047	0.182	−0.103	0.012	−0.038	−0.089	−0.013
RdATC‐212	227		0.000	0.000	0.000	0.000	0.000	0.010	0.000	0.000	0.000	0.000	
	233		0.366	0.236	0.294	0.359	0.272	0.300	0.313	0.304	0.188	0.170	
	236		0.000	0.009	0.000	0.000	0.009	0.000	0.010	0.000	0.000	0.000	
	239		0.071	0.164	0.022	0.033	0.097	0.040	0.021	0.020	0.375	0.348	
	242		0.143	0.227	0.294	0.239	0.123	0.200	0.188	0.216	0.073	0.125	
	245		0.027	0.009	0.033	0.033	0.061	0.030	0.042	0.020	0.031	0.080	
	248		0.009	0.009	0.033	0.033	0.000	0.010	0.010	0.000	0.010	0.054	
	251		0.339	0.309	0.315	0.294	0.386	0.390	0.354	0.353	0.146	0.107	
	254		0.045	0.036	0.011	0.011	0.053	0.020	0.052	0.078	0.104	0.107	
	257		0.000	0.000	0.000	0.000	0.000	0.000	0.010	0.010	0.052	0.009	
	260		0.000	0.000	0.000	0.000	0.000	0.000	0.000	0.000	0.021	0.000	
		*N*	56	55	46	46	57	50	48	51	48	56	
		*Na*	7	8	7	7	7	8 (1)	9	7	9 (1)	8	11 (2)
		*Rs*	6.713	7.233	6.879	6.888	6.719	7.603	8.542	6.73	8.832	7.732	7.653
		*He*	0.729	0.776	0.734	0.733	0.753	0.722	0.744	0.737	0.791	0.809	
		*Ho*	0.750	0.836	0.761	0.761	0.632	0.820	0.750	0.765	0.792	0.768	
		*Fis*	−0.029	−0.079	−0.038	−0.039^a^	0.162^a^	−0.137	−0.008	−0.038	−0.001	0.052^a^	−0.012
RdATC‐215	104		0.000	0.000	0.000	0.011	0.000	0.000	0.000	0.000	0.000	0.000	
	125		0.000	0.000	0.000	0.000	0.000	0.011	0.000	0.000	0.000	0.000	
	137		0.241	0.127	0.228	0.185	0.105	0.078	0.106	0.157	0.198	0.130	
	140		0.036	0.018	0.033	0.076	0.018	0.000	0.053	0.029	0.052	0.083	
	143		0.000	0.009	0.000	0.000	0.000	0.033	0.000	0.000	0.010	0.000	
	146		0.000	0.009	0.000	0.000	0.000	0.011	0.000	0.000	0.000	0.009	
	149		0.089	0.118	0.174	0.065	0.184	0.067	0.128	0.098	0.052	0.046	
	152		0.018	0.009	0.022	0.000	0.009	0.000	0.021	0.010	0.000	0.019	
	155		0.188	0.173	0.152	0.174	0.237	0.333	0.117	0.078	0.104	0.028	
	158		0.196	0.236	0.141	0.217	0.175	0.111	0.138	0.137	0.083	0.222	
	161		0.036	0.018	0.011	0.011	0.018	0.044	0.053	0.020	0.052	0.037	
	164		0.045	0.036	0.076	0.022	0.009	0.033	0.053	0.078	0.073	0.037	
	167		0.018	0.027	0.022	0.022	0.053	0.022	0.032	0.078	0.115	0.102	
	170		0.089	0.091	0.065	0.152	0.123	0.122	0.181	0.147	0.104	0.102	
	173		0.027	0.091	0.054	0.044	0.061	0.133	0.096	0.098	0.063	0.111	
	176		0.009	0.027	0.011	0.022	0.000	0.000	0.011	0.029	0.052	0.056	
	179		0.000	0.009	0.011	0.000	0.009	0.000	0.011	0.039	0.042	0.009	
	188		0.009	0.000	0.000	0.000	0.000	0.000	0.000	0.000	0.000	0.009	
		*N*	56	55	46	46	57	45	47	51	48	54	
		*Na*	13	15	13	12 (1)	12	12 (1)	13	13	13	15	18 (2)
		*Rs*	12.297	13.822	12.651	11.75	11.003	11.814	12.728	12.753	12.854	14.203	12.874
		*He*	0.854	0.872	0.869	0.861	0.853	0.838	0.897	0.902	0.907	0.893	
		*Ho*	0.929	0.873	0.913	0.804	0.877	0.911	0.915	0.922	0.771	0.833	
		*Fis*	−0.088	−0.001	−0.052	0.066^a^	−0.028	−0.089	−0.020	−0.022	0.151	0.067	−0.001
RdATC‐219	216		0.000	0.000	0.000	0.000	0.018	0.000	0.000	0.000	0.000	0.000	
	222		0.691	0.667	0.674	0.696	0.636	0.729	0.767	0.823	0.427	0.500	
	225		0.000	0.000	0.000	0.011	0.000	0.000	0.012	0.000	0.000	0.000	
	228		0.000	0.009	0.011	0.011	0.000	0.000	0.000	0.000	0.000	0.009	
	231		0.000	0.000	0.000	0.000	0.000	0.000	0.000	0.000	0.183	0.009	
	234		0.309	0.315	0.315	0.283	0.346	0.271	0.221	0.177	0.305	0.398	
	237		0.000	0.009	0.000	0.000	0.000	0.000	0.000	0.000	0.073	0.056	
	240		0.000	0.000	0.000	0.000	0.000	0.000	0.000	0.000	0.012	0.028	
		*N*	55	54	46	46	55	48	43	48	41	54	
		*Na*	2	4	3	4	3 (1)	2	3	2	5	6	8 (1)
		*Rs*	2	3.519	2.891	3.783	2.937	2	2.953	2	5	5.506	4.349
		*He*	0.431	0.461	0.451	0.441	0.480	0.399	0.366	0.295	0.694	0.593	
		*Ho*	0.364	0.556	0.435	0.370	0.509	0.458	0.372	0.313	0.488	0.333	
		*Fis*	0.158	−0.209	0.037	0.163	−0.062	−0.150	−0.016	−0.062	0.300^a^	0.440^b^	0.087
RdATC‐223	82		0.000	0.000	0.000	0.000	0.000	0.000	0.000	0.000	0.000	0.009	
	85		0.000	0.000	0.000	0.000	0.000	0.000	0.010	0.000	0.000	0.000	
	88		0.000	0.000	0.000	0.000	0.000	0.000	0.000	0.000	0.000	0.009	
	91		0.009	0.000	0.000	0.000	0.000	0.010	0.000	0.000	0.000	0.000	
	94		0.713	0.682	0.663	0.707	0.830	0.684	0.729	0.824	0.677	0.679	
	97		0.009	0.018	0.011	0.011	0.009	0.031	0.010	0.010	0.031	0.089	
	100		0.139	0.227	0.207	0.163	0.089	0.143	0.188	0.147	0.198	0.161	
	103		0.000	0.009	0.011	0.011	0.009	0.000	0.042	0.000	0.042	0.045	
	106		0.111	0.055	0.087	0.087	0.045	0.071	0.010	0.020	0.042	0.009	
	109		0.019	0.009	0.022	0.022	0.018	0.061	0.010	0.000	0.010	0.000	
		*N*	54	55	46	46	56	49	48	51	48	56	
		*Na*	6	6	6	6	6	6	7 (1)	4	6	7 (2)	10 (3)
		*Rs*	5.462	5.428	5.772	5.772	5.393	5.833	6.416	3.767	5.851	6.195	5.783
		*He*	0.464	0.484	0.515	0.471	0.303	0.508	0.436	0.303	0.503	0.508	
		*Ho*	0.500	0.473	0.457	0.522	0.339	0.551	0.458	0.275	0.438	0.464	
		*Fis*	−0.079	0.024	0.115^a^	−0.109	−0.122	−0.087	−0.053	0.094	0.132^a^	0.087	0.004
RdATC‐238	113		0.010	0.000	0.000	0.000	0.000	0.000	0.000	0.000	0.000	0.000	
	119		0.000	0.000	0.000	0.000	0.000	0.000	0.000	0.000	0.163	0.000	
	122		0.000	0.010	0.012	0.011	0.000	0.010	0.000	0.000	0.000	0.065	
	131		0.000	0.000	0.000	0.000	0.000	0.000	0.010	0.000	0.000	0.000	
	140		0.073	0.021	0.000	0.033	0.009	0.031	0.010	0.020	0.000	0.009	
	143		0.094	0.042	0.023	0.056	0.054	0.021	0.042	0.088	0.044	0.009	
	146		0.094	0.188	0.198	0.100	0.232	0.104	0.177	0.177	0.076	0.028	
	149		0.094	0.083	0.209	0.122	0.125	0.156	0.156	0.118	0.152	0.241	
	152		0.198	0.208	0.151	0.344	0.205	0.240	0.240	0.294	0.141	0.222	
	155		0.281	0.302	0.267	0.233	0.241	0.344	0.208	0.118	0.250	0.222	
	158		0.083	0.083	0.093	0.089	0.134	0.094	0.083	0.108	0.098	0.167	
	161		0.063	0.052	0.047	0.011	0.000	0.000	0.052	0.059	0.044	0.037	
	164		0.010	0.010	0.000	0.000	0.000	0.000	0.010	0.010	0.022	0.000	
	167		0.000	0.000	0.000	0.000	0.000	0.000	0.010	0.010	0.011	0.000	
		*N*	48	48	43	45	56	48	48	51	46	54	
		*Na*	10	10 (1)	8	9	7	8	11 (1)	10	10 (1)	9	14 (3)
		*Rs*	9.708	9.688	7.952	8.822	6.732	7.832	10.416	9.571	9.88	8.503	9.940
		*He*	0.848	0.820	0.821	0.799	0.817	0.787	0.840	0.839	0.857	0.817	
		*Ho*	0.771	0.813	0.791	0.578	0.607	0.375	0.833	0.745	0.739	0.556	
		*Fis*	0.092	0.009	0.037	0.279	0.258^b^	0.526^b^	0.008	0.113	0.139	0.322	0.180
RdATC‐263	177		0.010	0.000	0.000	0.000	0.000	0.000	0.000	0.010	0.000	0.000	
	183		0.000	0.010	0.022	0.000	0.000	0.011	0.000	0.010	0.031	0.063	
	186		0.000	0.000	0.011	0.000	0.000	0.000	0.000	0.000	0.010	0.018	
	189		0.000	0.000	0.000	0.000	0.000	0.000	0.000	0.000	0.021	0.089	
	192		0.481	0.343	0.435	0.424	0.219	0.160	0.141	0.122	0.208	0.214	
	195		0.000	0.000	0.022	0.011	0.009	0.000	0.033	0.020	0.010	0.063	
	198		0.298	0.353	0.315	0.359	0.632	0.649	0.598	0.714	0.208	0.116	
	201		0.173	0.275	0.163	0.174	0.132	0.160	0.217	0.112	0.458	0.357	
	204		0.039	0.020	0.011	0.022	0.009	0.021	0.011	0.010	0.031	0.045	
	207		0.000	0.000	0.022	0.011	0.000	0.000	0.000	0.000	0.010	0.018	
	210		0.000	0.000	0.000	0.000	0.000	0.000	0.000	0.000	0.010	0.000	
	213		0.000	0.000	0.000	0.000	0.000	0.000	0.000	0.000	0.000	0.009	
	216		0.000	0.000	0.000	0.000	0.000	0.000	0.000	0.000	0.000	0.009	
		*N*	52	51	46	46	57	47	46	49	48	56	
		*Na*	5	5	8	6	5	5	5	7	10 (1)	11 (2)	13 (3)
		*Rs*	4.787	4.767	7.75	5.772	4.439	4.857	4.89	6.485	9.392	10.323	7.095
		*He*	0.655	0.689	0.691	0.668	0.540	0.533	0.581	0.466	0.708	0.802	
		*Ho*	0.731	0.765	0.717	0.587	0.597	0.383	0.522	0.469	0.646	0.679	
		*Fis*	−0.117	−0.112	−0.039	0.122	−0.105	0.284	0.102	−0.007	0.088	0.155	0.035
RdATC‐28b	184		0.000	0.000	0.000	0.011	0.000	0.000	0.000	0.000	0.000	0.000	
	187		0.000	0.009	0.011	0.011	0.018	0.000	0.021	0.020	0.000	0.018	
	190		0.009	0.018	0.000	0.011	0.026	0.000	0.021	0.039	0.000	0.000	
	193		0.759	0.691	0.641	0.794	0.711	0.710	0.740	0.794	0.635	0.518	
	196		0.232	0.273	0.337	0.163	0.211	0.280	0.219	0.137	0.365	0.464	
	199		0.000	0.009	0.011	0.011	0.035	0.010	0.000	0.010	0.000	0.000	
		*N*	56	55	46	46	57	50	48	51	48	56	
		*Na*	3	5	4	6 (1)	5	3	4	5	2	3	6 (1)
		*Rs*	2.732	4.428	3.783	5.565	4.897	2.82	3.96	4.766	2	2.93	3.874
		*He*	0.373	0.452	0.480	0.347	0.453	0.422	0.409	0.352	0.468	0.521	
		*Ho*	0.339	0.509	0.478	0.348	0.333	0.380	0.438	0.314	0.396	0.536	
		*Fis*	0.092	−0.128	0.004	−0.002	0.265	0.100	−0.072	0.110	0.156	−0.029	0.050
All		*Na*	7.643	8.000	7.643	7.857	7.286	7.500	8.071	7.857	8.429	8.714	
		*Rs*	7.243	7.510	7.475	7.657	6.877	7.223	7.802	7.544	8.232	8.240	
		*He*	0.677	0.678	0.680	0.670	0.646	0.669	0.658	0.633	0.736	0.732	
		*Ho*	0.658	0.697	0.693	0.625	0.613	0.624	0.657	0.630	0.656	0.657	

a Significant departures from Hardy–Weinberg equilibrium at 5% level.

b Significant departures from Hardy–Weinberg equilibrium after sequential Bonferroni correction.
